# Supplementary material for: Rapid turnover of life-cycle-related genes in the brown algae
Source: Genome Biol. 2019 Feb 14;20:35. doi: 10.1186/s13059-019-1630-6 (PMC6374913; doi:10.1186/s13059-019-1630-6)
Supplement: Supplementary file 3 — Protein alignments, corresponding Gblocks html file and CDS sequences for the lineage conserved gametophyte-biased genes. (ZIP 2109 kb) [file 13059_2019_1630_MOESM3_ESM.zip › Con_GA_alignments/Ec-06_004530.aa_ali.fasta-gb.htm]

Ec-06\_004530.aa.fasta.trans.aa\_ali.fasta


## Gblocks 0.91b Results

Processed file: **Ec-06\_004530.aa.fasta.trans.aa\_ali.fasta**  
Number of sequences: **2**  
Alignment assumed to be: **Protein**  
New number of positions: **113** (selected positions are underlined in blue)

```
                         10        20        30        40        50        60
                 =========+=========+=========+=========+=========+=========+
Ec-06_004530     ---------------------------MSVQPAVEVRPAGQVGGKVTAMGSGATNFQYRK
MSTRG.17394.1    ATKEHLDGGNFGRKTVAGRGELGRASIMSVQPAVQVREAALADGKVVAMGSGGTNYQFRK
                                            #################################


                         70        80        90       100       110       120
                 =========+=========+=========+=========+=========+=========+
Ec-06_004530     VLNRESANAMKVISNIPRTQYVPGCFQRTLLCCWNMGTCKCCKCNCNCCISRKYLDAKMA
MSTRG.17394.1    SLNRQNADAVKIIYDIPRSKYTPGCCQN--LCSC----CKCCGDGSCPCECDAFEEVNKM
                 ###########################           ###########           


                        130       140       150       160       170       180
                 =========+=========+=========+=========+=========+=========+
Ec-06_004530     ADESTYTWVMENRLEYNNAMVVPKGQLVDFLCCNFQVADSAHVQYFDNDMFDYITVENSP
MSTRG.17394.1    AEESTYTWVMENRMEFSRPIVGLKDELCAACCCRFRVFDDIH------------------
                 ##########################################                  


                        190       200       210       220       230       240
                 =========+=========+=========+=========+=========+=========+
Ec-06_004530     TGCSRWCCGTPGDAIVFKTACCLCRTAKYCGIEKHRINVKTGDGKEISDIITTARDNARA
MSTRG.17394.1    ------------------------------------------------------------
                                                                             


                 
                 ===
Ec-06_004530     KMD
MSTRG.17394.1    ---
```

```
Parameters used
Minimum Number Of Sequences For A Conserved Position: 2
Minimum Number Of Sequences For A Flanking Position: 2
Maximum Number Of Contiguous Nonconserved Positions: 8
Minimum Length Of A Block: 10
Allowed Gap Positions: None
Use Similarity Matrices: Yes
```

```
Flank positions of the 3 selected block(s)
Flanks: [28  87]  [99  109]  [121  162]  

New number of positions in Ec-06_004530.aa.fasta.trans.aa_ali.fasta-gb:  113  (46% of the original 243 positions)
```
